# Outcomes of operative and nonoperative management of myotendinous Achilles tendon ruptures: a systematic review

**DOI:** 10.1186/s12891-025-08286-8

**Published:** 2025-01-20

**Authors:** Darius L. Lameire, Luca Ramelli, Mansur Halai, David Wasserstein, Sam Si-Hyeong Park

**Affiliations:** 1 Division of Orthopaedic Surgery, Department of Surgery , University of Toronto, Toronto, ON Canada; 2 Queen’s School of Medicine, Queen’s University, Kingston, ON Canada; 3 Division of Orthopaedic Surgery, St. Michael’s Hospital, Toronto, ON Canada; 4 Division of Orthopaedic Surgery, Women’s College Hospital, Toronto, ON Canada; 5 Division of Orthopaedic Surgery, Sunnybrook Health Sciences Centre, Toronto, ON Canada; 6 University of Toronto Orthopaedic Sports Medicine Program (UTOSM), Toronto, ON Canada

**Keywords:** Achilles, Myotendinous, Musculotendinous, Tear, Rupture, Treatment, Operative, Nonoperative

## Abstract

**Background:**

Achilles tendon ruptures are the most common lower extremity tendinous rupture. While there has been extensive research into the management of mid-substance Achilles tendon ruptures, there is a paucity of literature on the management of myotendinous Achilles tendon ruptures.

**Methods:**

The aim of this systematic review is to compile all available literature on the treatment of myotendinous Achilles tendon tears. A systematic search of Web of Science, Embase, and Medline databases was performed for all studies published from database inception to April 13, 2024. All publications addressing the treatment of myotendinous Achilles ruptures of all levels of evidence were included. The PRISMA Checklist guided the reporting and data abstraction. Descriptive statistics are presented.

**Results:**

A total of five studies with 70 patients were included for analysis. Sixty-seven patients underwent non-operative management with an average age ranging from 40.8 to 51.0 years. Three patients underwent operative management with ages of 16, 36, and 39. The majority of patients tore their Achilles tendon during sports. For nonoperatively treated patients, one group underwent immobilization for a total of 6 weeks and one study treated patients with functional rehabilitation. All patients were able to perform a single heel-raise, had good reported strength, and returned to work or sport. Nonoperative patients reported statistically significant improvements in subjective outcomes and high rates of satisfaction.

**Conclusion:**

Both nonoperative and operative management of myotendinous Achilles tendon ruptures demonstrated good outcomes after injury, although there is a limited amount of literature on this topic. Given that nonoperative treatment appears to yield good strength and return to activity, it may be preferred for the majority of patients. Operative management may be indicated in high level athletes. Imaging to determine the exact location of injury, quality of remaining tendon, and gap distance may further aid when considering treatment options. Higher level evidence studies are required to determine the optimal treatment of myotendinous Achilles tendon ruptures.

**Level of evidence:**

IV; Systematic review of Level IV-V studies.

**Supplementary Information:**

The online version contains supplementary material available at 10.1186/s12891-025-08286-8.

## Introduction

Achilles tendon ruptures are the most common lower extremity tendinous ruptures experienced by patients [1]. If not diagnosed and managed in a timely manner, this injury can result in significant morbidity and an inability to return to full athletic activity long-term [1]. The injury most commonly occurs in patients between the ages of 30 and 60 who participate in recreational sports on a sporadic basis (“the weekend warrior”) [2]. The sports that are most commonly associated with acute Achilles tendon ruptures include running, dancing, gymnastics, tennis, soccer, basketball, and American football [3]. These sports often require movements involving sudden forced plantar flexion of the foot, which is a common mechanism of Achilles tendon rupture [4]. Other mechanisms of injury include direct trauma and chronic degenerative tendinopathy. An increased risk of Achilles tendon ruptures has also been shown in patients with a history of prolonged corticosteroid use, overexertion, fluoroquinolone antibiotics, oral bisphosphonates, diabetes, end-stage renal disease, and hyperparathyroidism, although these ruptures tend to be more atraumatic in nature [3, 5–8].

The Achilles tendon typically ruptures two to six centimetres above the calcaneal insertion in the mid-substance of the tendon, as this area is where the blood supply to the tendon is diminished [9]. However, the next most common location of Achilles tendon rupture is at the myotendinous junction (MTJ, also known as the musculotendinous junction) [10]. The anatomic myotendinous junction is considered the zone of transition from the soleus to the Achilles tendon proper distally (as it combines with the aponeurosis of the two heads of the gastrocnemius) [11, 12]. The proportion of Achilles tendon ruptures occurring at the MTJ is unclear; however, one retrospective study that examined over 500 Achilles tendon rupture patients found that 6.7% of patients had a tear at the MTJ [13]. These MTJ Achilles tendon injuries have been reported to have increased calf swelling due to bleeding from the muscular portion of the tear [10], and are generally more difficult to diagnose clinically than their mid-substance counterparts. As such, radiographic evidence via ultrasound or MRI is often performed to confirm the diagnosis if an MTJ tear is suspected [7].

There is often debate surrounding the potential benefits versus risks of surgical intervention for Achilles tendon ruptures. Many studies have demonstrated good functional results and patient satisfaction with both nonoperative and operative modalities [8, 14–17]. Although conservative management has historically shown a higher re-rupture rate when compared to surgical interventions, the advancement of post-operative rehabilitation programs has decreased this risk significantly [16, 17]. However, the evidence on management of Achilles tendon ruptures has focused primarily on mid-substance tendon ruptures, and to our knowledge, no previous randomized controlled trials have reported on myotendinous Achilles tendon tears confirmed with advanced imaging or intraoperatively. As such, the optimal management of MTJ Achilles tendon ruptures is still not clear. The purpose of this systematic review is to compile all available literature on the treatment of myotendinous Achilles tendon tears. 

## Methods

This systematic review focused on all treatments of diagnosed acute myotendinous junction Achilles tendon ruptures. This review followed the Preferred Reporting Items for Systematic Reviews and Meta-Analysis (PRISMA) guidelines [18].

### Comprehensive search strategy

The databases of Web of Science, Embase, and Medline were searched systematically by two reviewers (D.L.L. and L.R.) for all studies published before April 13, 2024. A separate manual search was also completed to ensure no subsets of myotendinous tears were reported in studies focusing on all Achilles tendon tears. All publications assessing the management of myotendinous or musculotendinous junction Achilles ruptures/tears were included (complete search strategy can be found in Supplementary Digital Content [SDC] 1). Myotendinous and musculotendinous were used synonymously. The inclusion criteria consisted of: (1) diagnosed rupture/tear of the Achilles at the myotendinous junction (MTJ); (2) rupture/tear diagnosed by imaging or identified intra-operatively; (3) skeletally mature patients aged 16 years old or older; (4) acute management within 4 weeks of injury; (5) English language publications; and (6) all levels of evidence including case reports with single patients. The exclusion criteria consisted of: (1) Intrasubstance/mid-substance or insertional Achilles tendon ruptures/tears; (2) chronic Achilles tendon tears; (3) surgical technique publications without clinical outcomes, and (4) publications without any clinical outcomes reported. Given the wide anatomical variability between individuals in regards to the distance from the myotendinous junction to the calcaneal tuberosity, only studies that specifically reported myotendinous junction tears based off imaging or intraoperatively (not based on distances from the calcaneal tuberosity) were included [11, 19].

### Study screening

All study titles and abstracts were independently assessed by two authors (D.L.L. and L.R.) and included studies were advanced to full text review. Any disagreements were resolved by the senior author (S.S.P). The level of agreement between authors was evaluated by calculating a Kappa (κ) score [20]. The quality of studies was assessed using the Methodological Index for Non-randomized Studies (MINORS) [21]. For comparative studies, the maximum score is 24. For noncomparative studies, the maximum is 16. Scores for poor, fair, good, or excellent were determined by previously reported ranges [22].

### Data abstraction

Google Sheets (Alphabet, Inc) was used to abstract the data using predetermined tables by one reviewer, and another reviewer evaluated all abstracted data. Abstracted data included: number of patients, patient characteristics (age, gender, etc.), study information (title, authors, publication year, etc.), method of diagnosis of myotendinous junction Achilles tendon ruptures, follow-up period, treatment specifics in regards to operative and nonoperative management, and clinical outcomes. Level of evidence (LOE) was reported as specified by authors, or if not stated, based on the American Academy of Orthopaedic Surgeons (AAOS) Evidence-Based Practice Committee guidelines [23].

### Primary outcomes

The primary outcome of interest was to present the current management strategies reported for myotendinous achilles tears and their associated rehabilitation methods. This included both nonoperative rehabilitation strategies and operative management options.

### Secondary outcomes

The secondary outcomes consisted of: objective outcomes, rates of Achilles tendon re-rupture, patient reported outcome measures (PROMs), complications, and tendon elongation. Objective measures included heel raise strength, Achilles tendon resting angle (ATRA), and other strength outcomes. Heel raise strength was reported as the ability to perform the activity with a single-leg [24]. ATRA is a measure of the resting angle of the ankle and has been shown to be reliable in patients with Achilles tendon ruptures [25–27]. As per Carmont et al., [13] the ATRA is measured as “the angle between the long axis of the fibula and the line from the tip of the fibula to the head of the fifth metatarsal, measured using a standard 30 cm goniometer, with the patient lying prone and both knees flexed to 90 degrees. The relative ATRA is the difference between the ATRA on the injured side and the non-injured side.” Additionally, the heel-rise height index (HRHI) is determined as “patients perform a single leg heel-rise, and this is compared with function of the non-injured side to determine a limb symmetry index as a percentage" [28]. The heel-rise height is measured by asking the patient to stand on one leg on a 4 cm flat wooden block [13]. A tape measure is taped to the Achilles tendon so that the 10 cm mark is level with the block [13]. The patient is asked to perform a single heel-rise and the height raised is measured against the block” [13].

Patient reported outcome measures (PROMs) included the Achilles Tendon Total Rupture score (ATRS), the Foot and Ankle Ability Measure-Sports (FAAM-Sports), a visual analog scale for pain (VAS Pain), and satisfaction rates. ATRS is a questionnaire filled out by the patient with a maximum score of 100, which indicates full function and no symptoms [29]. The FAAM-Sports is a self-reported questionnaire that assesses the patient’s ability to do more difficult tasks deemed essential to sport [30]. The FAAM-Sports scale has an 8-item subscale, with a minimum score of 0 indicating unable to do any of the items to a maximum of 100 indicating no difficulty with any questioned tasks. VAS Pain is determined on a 10 cm scale, with 0 indicating no pain, and 10 indicating excruciating pain. To determine tendon elongation, Carmont et al. [13] considered it “to be a relative ATRA of more than ≥ 12° at 12-month evaluation. This relative ATRA is considered to be consistent with an acutely ruptured Achilles tendon [27]. The absence of a palpable gap and an ATRA consistent with rupture indicate tendon elongation.”

### Statistical analysis

Given the significant heterogeneity of the published papers, no data synthesis was performed for outcomes. Instead, outcomes were presented as a range of outcomes and in narrative summary fashion. Studies were sub-grouped based on operative and nonoperative treatment. Given the narrative fashion of reporting outcomes, no assessment of certainty of evidence was conducted.

## Results

**Fig. 1:**
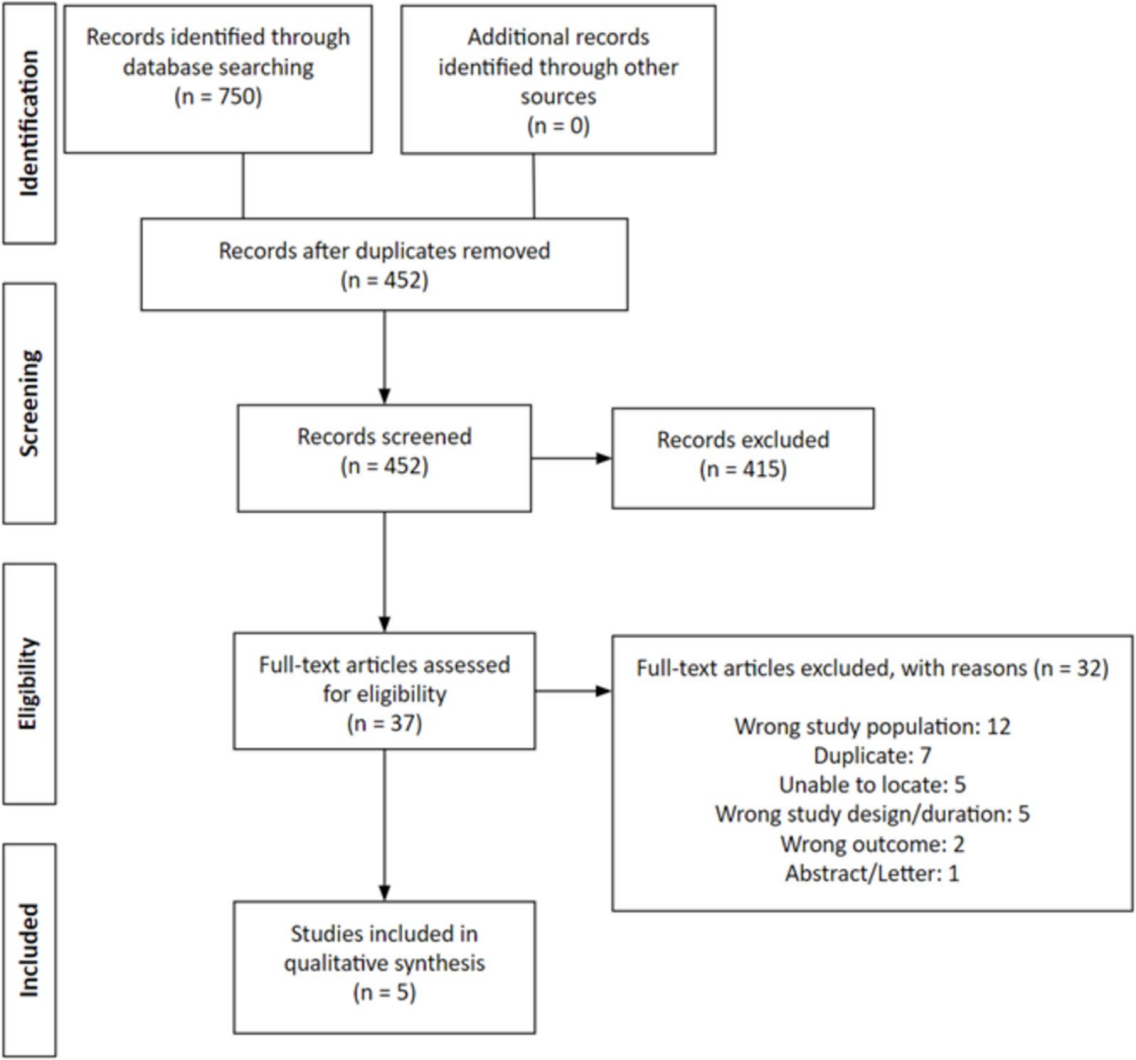
PRISMA diagram

The initial search identified 750 studies, with 452 studies remaining after duplicate removal (Fig. 1). After title and abstract review, 37 studies remained and were evaluated in full-text with a total of five studies included in this review [13, 24, 31–33]. There was substantial agreement between reviewers for title and abstract review (κ = 0.750; 95% CI, 0.625 – 0.876), and almost perfect agreement for full-text (κ = 0.874; 95% CI, 0.632 – 1.000). There was one retrospective cohort study [13] and one retrospective case series [24] (which were both level IV evidence), as well as three case reports [31–33] (which were level V evidence). All studies had good methodologic quality based on MINORS scoring. The comparative study [13] had a score of 18 and the average of noncomparative studies was 10.5 (SDC 2). Two studies assessed non-operative management of Achilles tears, with one comparative study and one case series [13, 24]. The comparative study assessed the outcome of nonoperative treatment of myotendinous and midsubstance Achilles tendon ruptures/tears [13]. The three case reports [31–33] all described patients undergoing operative management of myotendinous Achilles tendon ruptures via open surgical repairs.

### Patient demographics

There were a total of 70 patients included in this review. Sixty-seven patients underwent non-operative management (30 patients in one study [24] and 37 in another [13]), with an average age ranging from 40.8 to 51.0 years (Table 1). Three patients underwent operative management with ages of 16, 36, and 39. The female proportion of patients undergoing nonoperative management ranged from 10.8 to 30.0%, and there were two men and one female that underwent operative management. Follow-up average duration ranged from 12.0 to 40.5 months for nonoperative patients and the follow up was 6, 8, and 12-months for operative patients. Body Mass Index (BMI) was reported for two operative patients and was 34.0 and 37.6. Three studies [24, 31, 33] used MRI to diagnose the location of the Achilles tear, one study [13] used ultrasound, and one study [32] determined the location intra-operatively.

**Table 1:** Patient demographics

		Sex, n		**Side**		Age		Follow-up			
Author (Year)	Patients, n	Male	Female	Left	Right	Mean	SD (range)	Mean, mo	Range	BMI	Injury Activity
*Nonoperative*											
Ahmad (2013) [24]	30	21	9	15	15	40.8	NR (24–54)	40.5	23–81		Basketball 33%, Running 10%, Soccer 10%, Cross-training 6.7%, Baseball 3.3%, Martial arts 3.3%, Weightlifting 3.3%, Non-Athletic 20%
Carmont (2024) [13]	37	33	4	19	18	51	12.5 (NR)	12			Football 20%, Running 20%, Pushing vehicles 20%, Badminton 10%
*Operative*											
Gould (2021) [33]	1	1			1	36		6		34	Dodgeball
Shah (2009) [32]	1	1		1		39		8		37.6	Football
Valk (2023) [31]	1		1	1		16		12			Cheerleading

*BMI* Body mass index, *NR* Not reported, *SD* Standard deviation

Sports/athletic participation was the most common activity reported during injury (Table 1). One publication [33] reported that the patient was using a selective androgen receptor modulator (SARM) prior to Achilles rupture, and this same patient tore their opposite Achilles tendon on postoperative day four when using it as their weightbearing leg. This second tear was located just distal to the myotendinous junction.

### Rehabilitation protocol

Full rehabilitation protocols for each study can be found in Table 2. Four studies [24, 31–33], including all operative patients, had a period of initial non-weightbearing (NWB). For the non-operative patients initially immobilized, they were progressed to weightbearing as tolerated (WBAT) at four weeks. For operative patients, they were progressed to WBAT between five to seven weeks postoperatively. The second nonoperative study made their patients touch-weightbearing (TWB) initially and appeared to progress their patients to WBAT by 6 weeks (although their reported protocol does not specifically state this).

**Table 2:** Rehabilitation protocol

Author (Year)	Rehabilitation Protocol	WB Status
*Nonoperative*		
Ahmad (2013) [24]	1. Achilles boot (Bledsoe Brace Systems, Grand Prairie, TX) with three 1-inch wedges at the heel, non-weight-bearing on their affected leg for 3 weeks 2. At 3 weeks, patients were allowed to progressively bear weight on their injured leg in their Achilles boots. A physical therapy program for Achilles tendon strengthening was instituted after the initial 3 weeks, with all therapy performed out of the boot full weight bearing. They were also instructed to remove a single 1-inch wedge from the boot after every week until their ankle was plantigrade 3. At 6 weeks, patients were allowed to taper out of their Achilles boots and increase their level of activity as tolerated 4. Between 6 and 12 weeks, patients were allowed to return to work without restrictions so long as their pain and function had improved	Weeks 1–3: NWB Weeks 4 + : WBAT
Carmont (2024) [13]	1. All patients were placed into an equinus functional brace made from synthetic casting material in plantar flexion. Patients were permitted to mobilise touch weight-bearing on the metatarsal heads as tolerated using elbow crutches 2. After two weeks, the posterior half of the cast was removed leaving the anterior shell in situ. This restricted dorsiflexion but permitted some active plantar flexion. At this two-week time point, active plantar flexion, inversion, and eversion exercises were commenced, but active dorsiflexion was avoided 3. After the six-week time point, the functional brace was discontinued and patients were given a 1.5 cm in-shoe heel wedge for the next 6 weeks and referral for physiotherapy for gait retraining and a progressive strengthening exercise programme	Weeks 1–6: TWB Weeks 6 + : WBAT
*Operative*		
Gould (2021) [33]	1. Postoperatively, the patient was placed into a well-padded short leg splint in 20° of plantarflexion. They were instructed to remain non-weightbearing on his right lower extremity for 4 weeks 2. He was scheduled to return to the outpatient clinic at 2 weeks postoperatively for a wound check, with plans to transition at that time from the splint to a plantigrade controlled ankle movement (CAM) walker boot 3. The patient completed a 3-month course of formal physical therapy after which he resumed his own workout regimen	Weeks 1–4: NWB Week 5 + : WBAT
Shah (2009) [32]	1. Non-weightbearing immediately post-op in a gravity-equinus well-padded splint 2. One week after the operation, the patient was placed in a short-leg gravity equinus cast and allowed toe-touch weight bearing with crutches 3. Three weeks postoperatively he was placed in an ankle-stabilizing walking boot with a 1/4-inch heel lift, remaining toe-touch weight bearing but allowing gentle range of motion exercises twice a day 4. At 6 weeks he was started on isotonic exercises with full range of motion and was allowed weight bearing as tolerated in the boot 5. At 9 weeks postoperatively the boot was discontinued, and a full physical therapy protocol was initiated 6. At 6 months he was allowed to run 7. At 8 months he made a full recovery and returned to sports	Week 1: NWB Weeks 2–5: TWB Week 6 + : WBAT
Valk (2023) [31]	1. Immediately postop, she was placed into a well-padded short-leg splint in maximum equinus to unload the repair 2. At her 2-week postoperative visit, she was transitioned from her splint into a non–weight bearing short-leg cast. She was able to be passively dorsiflexed to 20° shy of neutral 3. At 6 weeks, she was taken out of her cast and kept non–weight bearing in a controlled ankle motion boot 4. The patient was seen at 7 weeks and physical therapy and full weight bearing were initiated 5. At 9 weeks, she was able to passively dorsiflex to 10° and was transitioned to a shoe 6. At 4 months, she regained full ankle range of motion with 20° dorsiflexion	Weeks 1–6: NWB Week 7 + : WBAT

*NWB* Non-weightbearing, *TWB* Touch weightbearing, WB Weightbearing, *WBAT* Weightbearing as tolerated

### Operative technique

All three case reports of operative repair used a posterior open approach (SDC 3). All directly repaired the tendon at the myotendinous junction using sutures. Two studies [31, 33] used FiberWire (Arthrex) and one study [32] used Ethibond. One study [31] had their repair augmented using a bioinductive collagen patch (Regeneten, Smith & Nephew).

### Objective outcomes

Objective outcomes were limited in the included studies of this review (Table 3). One study [13] reported relative ATRA at 9- and 12-months post-injury and HRHI at 12-months for nonoperatively treated patients. This study was comparative between myotendinous and mid-substance Achilles tendon ruptures. For myotendinous tears, at 9-months they found a statistically significant decrease (better outcome) in ATRA than for midsubstance tears (*p* = 0.006), and again at 12-months (*p* = 0.019). At final follow-up, there was only an average of 5.5 degrees less resting dorsiflexion on the affected limb compared to the contralateral. Additionally, they found a statistically significant difference in HRHI for myotendinous tears (*p* = 0.019). The heel rise index on the affected limb was on average 79% compared to the contralateral.

**Table 3:** Objective outcomes

	ATRA				HRHI		Strength
	9mo Follow-up		12mo Follow-up				
Author (Year)	Mean, deg	SD	Mean, deg	SD	Mean, %	SD	
*Nonoperative*							
Ahmad (2013) [24]							No weakness, all patients able to perform 20 repetitive single-leg heel raises
Carmont (2024) [13]	-6.9	4.6	-5.5	5	79	25	All patients were able to perform single heel rise on affected side
*Operative*							
Gould (2021) [33]							The patient had returned to powerlifting at approximately 80% strength compared with his preinjury state
Shah (2009) [32]							Able to easily perform single heel-raise at 4 months
Valk (2023) [31]							Able to perform single heel-raise at 5 months

*ATRA* Achilles tendon resting angle, *Deg* degree, *HRHI* heel raise height index, *Mo* months, *SD* Standard deviation

Four studies [13, 24, 31, 32] (two studies that treated nonoperatively and two studies treated operatively) reported that all patients with myotendinous Achilles tendon ruptures/tears were able to perform a single heel-raise. One study [33] reported that an operatively treated patient was able to return to powerlifting at ~ 80% preinjury strength (this patient had bilateral tears).

### Return to work/sport

One study [24] assessing non-operative management reported that all patients were able to return to work at full capacity at an average of 11.8 weeks (range, 8 to 22 weeks) after injury. For operative studies, one patient [32] returned to full sport (football) at eight months post operation and one patient [31] returned to full competitive gymnastics at 12 months. The other operative patient [33] returned to powerlifting at 80% strength.

### Patient reported outcome measures

The reported PROMs were limited in the included studies (Table 4). Two studies [13, 33] reported ATRS scores at follow-up. One study assessing nonoperative management found a mean ATRS for myotendinous junction Achilles tendon tears was 83.6. The other study reporting on an operative patient that had bilateral Achilles tendon tears (one myotendinous tear and one tear just distal to the myotendinous junction) and reported a score of 60 bilaterally [33]. One study [24] reported FAAM-Sports, VAS Pain, and satisfaction rates for nonoperatively treated patients. The mean immediately post-injury FAAM-Sports score was 20 which improved to 95.2 (p < 0.05) at final follow-up. VAS pain improved from 8.2 post-injury to 1.3 (p < 0.01) at final follow-up. They also reported that 76.7% of patients rated their outcome as excellent, 20% reported it as good, and 3.3% reported their outcome was fair.

**Table 4:** Patient reported outcome measures

	ATRS		FAAM-Sports				VAS Pain				Satisfaction		
	Follow-up		Initial		Follow-up		Initial		Follow-up		Excellent	Good	Fair
Author (Year)	Mean	SD	Mean	Range	Mean	Range	Mean	Range	Mean	Range	%	%	%
*Nonoperative*													
Ahmad (2013) [24]			20.2	(31–89.3)	95.2*	(66.7–100)	8.2	(2–10)	1.3*	(0–5)	76.7	20	3.3
Carmont (2024) [13]	83.6	3.5											
*Operative*													
Gould (2021) [33]	60												

*ATRS* Achilles tendon total rupture score, *FAAM* Foot and Ankle Ability Measure, *VAS* Visual analog scale

### Complications

There was one reported possible superficial infection in the operative case reports [31], and they were started on cephalexin as prophylaxis and did not have any issues. No re-ruptures were reported for either operative or nonoperative management of myotendinous Achilles tendon tears. In the comparative study [13] between nonoperatively treated myotendinous and midsubstance tears, they reported no re-ruptures in the myotendinous group, but two patients (8.3%) in the midsubstance group re-ruptured. Otherwise, for the myotendinous tears group, they reported one deep-vein thrombosis and one case of adhesions.

## Discussion

To our knowledge, this is the first systematic review to assess the management of myotendinous junction Achilles tendon ruptures. This study was undertaken given the clinical concern, in our experience, of increasing patients unsatisfied with nonoperative management of their myotendinous Achilles tendon tears that have healed elongated; decreasing their push off strength, altering their gait, and impacting their satisfaction. The majority of studies in this review assessed patients with myotendinous Achilles tendon ruptures undergoing nonoperative rehabilitation, with only three case reports on operative management. Regardless of treatment type, all patients were reported to have good strength as evident by their ability to perform a single-heel raise, and were able to return to sport or work. Patients also reported improved subjective outcomes including reduced pain, improved ability to perform sport activities, and high rates of satisfaction.

Nonoperative management of Achilles tendon ruptures may be influenced by the location of the tendon rupture. Functional rehabilitation has been shown to be an effective treatment option for midsubstance Achilles tears [17], however, there has been limited research on the best rehabilitation protocol for nonoperative management of myotendinous tears. In this review, one study [24] treated patients with classical immobilization and another study [13] treated patients with more modern functional rehabilitation. In the study by Carmont et al. [13], the authors compared nonoperative management of myotendinous Achilles tendon ruptures to that of midsubstance Achilles tendon ruptures using functional rehabilitation. They found that with their rehabilitation protocol (shown in Table 2), patients with myotendinous Achilles tendon ruptures had no objective tendon elongation (definition in the Methods section) at final follow-up. In comparison, they found that five patients with midsubstance Achilles tendon ruptures unfortunately experienced tendon elongation. Tendon elongation can significantly impact a patient’s plantarflexion power and overall satisfaction [34, 35]. All patients in the myotendinous group were able to do a single-leg heel raise and had on average 79% the heel-raise ability (HRHI) compared to their contralateral side. In comparison, two patients with midsubstance tears were unable to do a single heel-raise (although they could not do one on their uninjured side either) and their HRHI was 59%. Based on the findings reported by Carmont et al. [13], myotendinous junction Achilles tendon ruptures may be more amenable to nonoperative functional rehabilitation with less risk of tendon elongation and weakness compared to midsubstance Achilles tendon ruptures.

Additionally, Ahmad et al. [24] found full healing of the Achilles MTJ in all 30 patients studied and concluded that “nonsurgical treatment of myotendinous Achilles ruptures results in a high rate of myotendinous healing and improved patient function” [24]. This may be due to the innate regenerative capabilities of the myotendinous junction, along with increased healing due to improved blood flow compared to the tendon proper [11, 36]. A recent in-vivo mice study by Yamamoto et al. [37] examined the regeneration process of the MTJ after injecting collagenase (simulating damage/tearing of the MTJ) [37]. They found that MTJ regeneration occurs along with regeneration of the muscle. They also found that after application of collagenase, the MTJ significantly shortened, however over time, it recovered in length [37]. This study further demonstrated that MTJ injuries have the potential of regenerating and remodeling, which may be why outcomes of nonoperative management of MTJ have good rates of healing and patient function.

The majority of patients in this review sustained their myotendinous Achilles tendon ruptures during sport. The Achilles tendon is comprised of the soleus and the medial and lateral heads of the gastrocnemius [24]. The soleus originates from the posterior aspect of the tibia and only crosses the ankle, whereas the gastrocnemius originates from the posterior aspect of the femur and crosses both the knee and the ankle [32, 38]. When the Achilles tendon is stretched, there may be differential stresses applied to the gastrocnemius and soleus depending on the position of the foot and knee, and may predispose the Achilles tendon to tear at the myotendinous junction versus midsubstance [32]. Interestingly, the comparative study between midsubstance and myotendinous tears [13], found that 29% of patients with midsubstance tears sustained their injury when only walking with none reported for myotendinous tears. This is a relatively low energy mechanism injury compared to the more active sports reported in their patients with myotendinous tears. This may be due to the differential load stressed on the posterior calf muscles during certain exercises that my pre-dispose patients to tear the myotendinous or the midsubstance portion. Although there are no studies published on this, it is possible that when myotendinous junction tears occur, they occur more preferentially in sports/activities with a more predominant athletic stance (knees bent) than when patients are at lower demand and knees are straighter (walking).

In terms of surgical management of myotendinous Achilles tendon ruptures, many different options have been described. Giordano et al. [10] performed a retrospective review of all patients at their institution that underwent operative management of an Achilles tendon rupture. This study was excluded from our review as there were no clinical outcomes reported for the patients. For myotendinous junction ruptures, they found that 57.1% (eight patients) had standard end-to-end repairs of the tendon and that 42.9% (six patients) had an augmented repair (defined as “using an isolated flexor hallucis longus (FHL) transfer, FHL transfer plus end-to-end repair, or allograft”). In our systematic review, we included three case reports of patients that underwent operative management of their myotendinous junction Achilles tendon ruptures. All patients underwent direct end-to-end tendon repairs, with one patient having an additional adjunct. The patient with an adjunct was a 16-year-old female who was an elite gymnast [31]. The authors discussed the patient’s case with “local and national experts in the Subspecialties of Sport and Foot & Ankle” and considered the use of a Regeneten (Smith & Nephew), a bioinductive collage patch, to augment the repair. Intraoperatively, they reduced the tendon with “excellent” approximation but decided to augment with the bioinductive collagen patch due to elite level of the athlete and the myotendinous location of the rupture. Postoperatively, she was able to return to full sport and competition but did have a superficial wound concern and was given cephalexin prophylactically. MRI obtained between 6- to 7-months after surgery showed tendon healing with increased thickness. The authors concluded that for high level athletes and/or tears of the myotendinous junction, bioinductive collagen patches may have a role in augmentation.

Locating the site of Achilles tendon rupture is important, as there may be different outcomes based on common rupture sites (as measured in relation to the calcaneal insertion). Studies by Bäcker et al. [39] and Park et al. [40] found that the average distance from rupture to calcaneal insertion was 5.86 ± 2.66 cm and 6.4 ± 1.5 cm, respectively. This was somewhat contradictory to previous reports indicating that common tears occurred closer to the calcaneal insertion, from 2–6 cm [9]. Cao et al. [9] compared the surgical outcomes of 117 patients that had surgical management of Achilles tendon ruptures based on location of tear [9]. They found the average rupture site was 4.5 ± 1.3 cm from the calcaneal insertion. The authors also found that those with more distal Achilles tendon tears (< 4.5 cm) had more satisfactory postoperative outcomes (77% of patients had an ATRS > 80), than those with more proximal tears (≥ 4.5 cm; 56% of patients had an ATRS > 80) [9]. This study was excluded from the present review as they did not specifically report and compare MTJ tears, although those that were more proximal may have in fact been, or were very close to, the MTJ. This may indicate that more proximal tears in the MTJ may have worse surgical outcomes compared to mid-substance tears.

The exact location of Achilles tear, gap distance, and quality of surrounding/remaining tendon/fascia can have significant clinical implications. Cao et al. [9] used ultrasound to determine the exact location of the tear, as the clinical examination can be excellent in determining if there is disruption of the Achilles tendon but can be unreliable in the location and gap distance [33]. A survey of Orthopaedic surgeons found that there was confusion regarding the exact location of the myotendinous junction. The majority of surgeons estimated the level of the myotendinous junction to be higher than it was [11]. Therefore, surgeons may offer surgery on tears of at MTJ, although they believe they will be operating on a midsubstance tear based on clinical examination solely. If more proximal tears and tears of the MTJ fair worse surgically than those treated nonoperatively [9], it is important to know exactly where the tear is and its relation to the MTJ, to better counsel patients on the expectations of operative vs nonoperative management. MRI is effective for assessing the Achilles tear location; however it is expensive and can be less available to patients relative to other imaging modalities [41]. Ultrasound on the other hand, is readily available and relatively inexpensive. There may be concern that ultrasound would be inadequate but recent studies have shown that ultrasound, with a well-trained ultrasound technologist, can be just as reliable as MRI for determining tear location and gap size [41]. Therefore, given the availability of ultrasound, this may be a great modality to examine all Achilles tendon tears to better counsel patients on expected operative outcomes.

### Limitations

The main limitation of this study is the limited number of studies assessing the outcomes of nonoperative and operative treatment of myotendinous Achilles tendon ruptures. Nevertheless, this review is an up-to-date and comprehensive review of all the literature, which creates a foundation for clinical treatment and further research. Another limitation was the level of evidence of the included studies was low, with all studies being level IV or lower. With operative management, there were only three case reports (level V evidence), which may reflective the rarity of operative management of this injury. Lastly, one study [13] included both partial and full myotendinous Achilles tendon ruptures (excluded isolated tears of the medial gastrocnemius), which can alter the strength outcomes, as partial tears may have less risk of tendon elongation.

## Conclusion

Both nonoperative and operative management of myotendinous Achilles tendon ruptures demonstrated good outcomes after injury, although there is a limited amount of literature on this topic. Given that nonoperative treatment appears to yield good strength and return to activity, it may be preferred in the majority of patients. Operative management may be indicated in high level athletes. Imaging to determine the exact location of injury, quality of tendon, and gap distance may further aid when considering treatment options. Higher level evidence studies are required to determine the optimal treatment of myotendinous Achilles tendon ruptures.

## Supplementary Information

Supplementary Material 1.

## Data Availability

All data and materials are available publicly based on previous publications.

## Abbreviations

AAOS: American Academy of Orthopaedic Surgeons
ATRA: Achilles Tendon Resting Angle
ATRS: Achilles Tendon Total Rupture Score
BMI: Body Mass Index
CAM: Controlled Ankle Movement
FAAM: Foot and Ankle Ability Measure
HRHI: Heel-Rise Height Index
LOE: Level of Evidence
MINORS: Methodological Index for Non-randomized Studies
MRI: Magnetic resonance imaging
MTJ: Myotendinous junction
NWB: Non-weightbearing
PROMs: Patient Reported Outcome Measures
PRISMA: Preferred Reporting Items for Systematic Reviews and Meta-Analysis
SARM: Selective Androgen Receptor Modulator
SDC: Supplementary Digital Content
TWB: Touch-weightbearing
WB: Weightbearing
WBAT: Weightbearing as Tolerated
VAS: Visual Analog Scale
