# Supplementary material for: Outcomes of operative and nonoperative management of myotendinous Achilles tendon ruptures: a systematic review
Source: BMC Musculoskelet Disord. 2025 Jan 20;26:71. doi: 10.1186/s12891-025-08286-8 (PMC11744937; doi:10.1186/s12891-025-08286-8)
Supplement: Supplementary file 1 — Supplementary Material 1. [file 12891_2025_8286_MOESM1_ESM.docx]

Supplementary Digital Content 1: Search Strategy

| Medline | Embase | Web of Science |
| --- | --- | --- |
| 1 musculotend*.mp. (1773)  2 exp Myotendinous Junction/ or myotend*.mp. (1013)  3 1 or 2 (2748)  4 exp Achilles Tendon/ or achill*.mp. (18122)  5 3 and 4 (213) | 1 musculotend*.mp. (2208)  2 exp Myotendinous Junction/ or myotend*.mp. (1287)  3 1 or 2 (3423)  4 exp Achilles Tendon/ or achill*.mp. (29875)  5 3 and 4 (280) | 1 musculotendinous or myotendinous and achilles (257) |

Supplementary Digital Content 2: Study Characteristics

| **Author (Year)** | **Title** | **Journal** | **Published Year** | **Recruitment Period** | **Country** | **Study Design** | **LOE** | **Intervention Group** | **Comparator Group** | **MINORS** |
| --- | --- | --- | --- | --- | --- | --- | --- | --- | --- | --- |
| Ahmad (2013) | Treatment of myotendinous Achilles ruptures | Foot Ankle Int. | 2013 | November 2005 to May 2011 | USA | Retrospective case series | IV | Non-operative |  | 12 |
| Carmont (2024) | Musculotendinous ruptures of the achilles tendon had greater heel-rise height index compared with mid-substance rupture with non-operative management: A retrospective cohort study | J ISAKOS | 2024 | February 2009 to August 2023 | UK | Retrospective cohort | IV | Non-op: Myotendinous | Non-op: Midsubstance | 18 |
| Gould (2021) | Asynchronous Bilateral Achilles Tendon Rupture with Selective Androgen Receptor Modulators: A Case Report | JBJS Case Connect | 2021 | NA | USA | Case report | V | Open surgical repair |  | 10 |
| Shah (2009) | A unique myotendinous avulsion injury of the Achilles tendon | Current Orthop Practice | 2009 | NA | USA | Case report | V | Open surgical repair |  | 10 |
| Valk (2023) | Bioinductive Collagen Implant Augmentation for Myotendinous Achilles Rupture in a Teenage Competitive Gymnast: A Case Report | JBJS Case Connect | 2023 | NA | USA | Case report | V | Open surgical repair |  | 10 |

Supplementary Digital Content 3: Operative Techniques

| **Author (Year)** | **Technique** | **Sutures** | **Adjuncts** |
| --- | --- | --- | --- |
| Gould (2021) | Achilles tenorrhaphy through an open posteromedial approach. | No. 2 FiberWire sutures were passed through the proximal and distal tendon stumps in a running locked Krackow configuration and hand-tied over the rupture site. |  |
| Shah (2009) | A curvilinear incision was made proximally along the posterior calf. The tendon was repaired, attaching the small avulsed portion of the lateral tendon back to the main portion of the Achilles tendon and then securing the proximal portion of the tendon back down to the gastrocnemius muscle belly. | No. 2 Ethibond suture was used in the distal portion of the repair. For the proximal portion of the repair No. 2 Ethibond sutures were used in a running fashion up the medial and lateral sides, with further reinforcement in the central portion using 2-0 Vicryl (Ethicon) sutures |  |
| Valk (2023) | A longitudinal incision was made over the posteromedial aspect of the Achilles tendon with dissection through the superficial and deep fascia. End-to-end repair with a locking suture technique was performed in an open fashion using high tensile suture. The tendon was reduced in the dependent equinus position. The tendon was then tubularized with absorbable suture, then the frayed ends repaired over top in a pants-over-vest fashion. | No. 2 and No. 5 FiberWire (Arthrex) for repair, the tendon was then tubularized with absorbable suture. The wound was closed in layers with a running subcuticular closure and skin glue (Dermabond; Ethicon). | The decision was made intraoperatively to augment the repair using bioinductive collagen patch (REGENETEN) It was sewn over the repair with absorbable suture (2-0 Vicryl). |
